# Polyphosphate in Bone Tissue Engineering: From Molecular Mechanisms to Material Design

**DOI:** 10.3390/jfb17080422

**Published:** 2026-08-21

**Authors:** Zhangling Nie, Bingqiang Lu, Valentina K. Krut’ko, Anatoly I. Kulak, Feng Chen

**Affiliations:** 1 Shanghai Key Laboratory of Craniomaxillofacial Development and Diseases, Shanghai Stomatological Hospital & School of Stomatology, Fudan University, Shanghai 201102, China; zhanglingnie@fudan.edu.cn (Z.N.); bingqianglu@fudan.edu.cn (B.L.); 2 Institute of General and Inorganic Chemistry, National Academy of Sciences of Belarus, 220072 Minsk, Belarus; tsuber@igic.bas-net.by

**Keywords:** polyphosphate, bone tissue engineering, osteogenic differentiation, biomaterials, bone regeneration

## Abstract

Polyphosphate (PolyP) is an inorganic polymer composed of orthophosphate units linked by high-energy phosphate anhydride bonds, widely found in various organisms from bacteria to mammals. In recent years, PolyP has attracted widespread attention in the field of bone tissue engineering due to its unique biological characteristics, possessing both osteoinductive activity and metabolic energy supply functions. This article systematically reviews the molecular structure, physicochemical properties, and multiple mechanisms by which PolyP promotes osteogenic differentiation, as well as biomaterial design strategies based on PolyP. PolyP can synergistically promote osteogenic differentiation through multiple mechanisms, including by acting as a phosphate donor, providing metabolic energy, regulating signaling pathways such as Wnt/β-catenin, and modulating the osteoprotegerin/receptor activator of nuclear factor κB ligand (OPG/RANKL) balance. In terms of material design, PolyP can form nano/microparticles with metal ions such as Ca^2+^, Sr^2+^, and Mg^2+^ and can also be compounded with polymers to construct various forms such as hydrogels, bone cement, and three-dimensional (3D)-printed scaffolds. Preclinical studies have shown that PolyP-incorporated materials exhibit excellent osteogenic performance and biocompatibility in bone defect repair, and preliminary clinical studies have also confirmed its feasibility. This article aims to provide a comprehensive overview of the current applications of PolyP-incorporated materials and delineate future directions, challenges, and necessary pathways for their clinical translation.

## 1. Introduction

The repair of bone defects caused by trauma, tumor resection, infection, or congenital malformation remains a major clinical challenge [1]. Although autologous bone transplantation is regarded as the gold standard, it encounters problems such as limited donor sources and secondary damage [2]. Therefore, the development of bone repair substitutes with excellent osteogenic activity is a research hotspot in the field of tissue engineering. In recent years, artificial bone substitutes have been widely used in the repair of large-volume bone defects due to their advantages such as strong designability, wide availability and low immunotoxicity [3]. However, existing materials still face key bottlenecks such as insufficient mechanical strength, mismatch between degradation rate and new bone formation, and limited osteogenic induction activity [4,5]. Moreover, in the context of complex pathological backgrounds such as osteoporosis, infection or old age, the deterioration of the bone microenvironment further exacerbates the difficulty of repair [6,7]. Therefore, it is urgent to develop new bone substitutes that combine bioactivity, structural adaptability and functional tunability.

Polyphosphate (PolyP) is a naturally occurring biopolymer composed of chains of tens to hundreds of orthophosphate residues linked by high-energy phosphoanhydride bonds, widely present in organisms from bacteria to humans [8]. As early as the 1960s, researchers noted the role of PolyP in tissue mineralization [9]. While early studies primarily focused on its functions in energy storage and microbial stress responses, its key regulatory roles in the mineralized tissues of higher organisms have only been gradually revealed in recent years [10]. PolyP naturally exists in mammalian platelets, osteoblasts, and body fluids, playing a significant role in bone metabolism [11,12]. Compared with traditional bone repair materials, PolyP offers unique advantages: first, as a phosphate donor, it provides raw materials for hydroxyapatite (HA) formation [13]; second, the hydrolysis of its high-energy phosphoanhydride bonds can support the generation of adenosine triphosphate (ATP), thereby meeting the energy demands of bone regeneration [14]; third, PolyP possesses osteoinductive activity, capable of regulating the expression of osteogenesis-related genes [15]. These properties make PolyP an ideal candidate for constructing next-generation bone repair materials.

Based on the fundamental biological characteristics of PolyP well-documented in previous reviews, this review highlights the critical relationship between its inherent structural properties and biomaterial design applications. This review explicitly delineates the structure–function relationship of PolyP and its multifaceted osteogenic mechanisms, specifically evaluating its roles as a phosphate donor, an extracellular metabolic energy source, and a modulator of immune–bone coupling signaling pathways. Furthermore, we critically analyze current PolyP-incorporated biomaterial design strategies, ranging from metal ion complexes and polymer composites to three-dimensional (3D)-printed scaffolds and smart coacervates, and assess their specific efficacies in bone tissue engineering. The distinct objective of this review is to identify critical material design bottlenecks and to inspire structured pathways built upon existing research, practical experience and fundamental theories to overcome current challenges in PolyP-incorporated bone substitutes.

## 2. Molecular Structure and Basic Functions of PolyP

### 2.1. Chemical Structure and Physicochemical Properties

**Figure 1:**
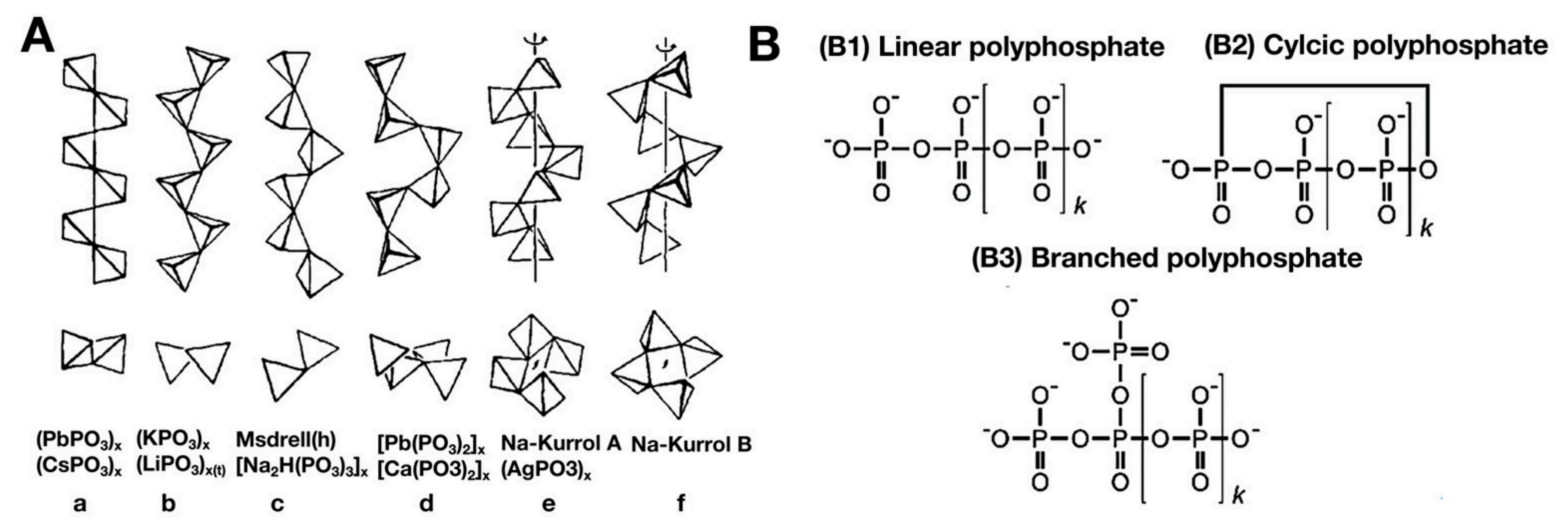
(**A**) The typical types of anion chains in high-molecular-weight crystalline polyphosphate (PolyP). Above: vertical projections; below: projections along the chain axis. (Adapted with permission from Ref. [20]. Copyright **2003** John Wiley and Sons). (**B**) The molecular structures of linear PolyP (**B1**), cyclic PolyP (**B2**), and an exemplary branched PolyP (**B3**) are shown. In part (**B3**), the branching point(s) can be elsewhere, and the chain length of the side chain(s) can vary. k can range between 0 and ∼1000, 1 and ∼10, and 0 and ∼1000 for parts (**B1**), (**B2**), and (**B3**), respectively. (Adapted with permission from Ref. [18]. Copyright **2020** American Chemical Society).

PolyP is a class of inorganic biopolymers consisting of tens to hundreds of orthophosphate residues linked by high-energy pyrophosphate bonds [16]. Its basic structural unit is phosphate (PO_4_^3−^), and adjacent units form pyrophosphate bonds in the form of P–O–P [17]. PolyP is structurally classified into three main forms: linear, cyclic, and branched, which exhibit significant differences in their physicochemical properties [18]. Cyclic PolyP is relatively stable in aqueous solutions, but there is no evidence indicating a specific biological function; the cyclic structures found in cell extracts are mostly byproducts of the spontaneous hydrolysis of linear chains. Branched PolyP lacks resonance stabilization due to the presence of “tertiary” phosphorus atoms coordinated with three adjacent phosphorus atoms; according to the “antibranching rule,” it is extremely unstable in aqueous environments and highly susceptible to rapid hydrolytic cleavage. Consequently, linear PolyP predominates as the active functional form in biological systems. At physiological pH, each phosphate unit carries one negative charge, making PolyP one of the natural biopolymers with the highest negative charge density [19]. This property enables it to interact electrostatically with various cations (Ca^2+^, Mg^2+^, Sr^2+^, etc.) to form salts or complexes. Crystalline linear PolyP demonstrates a high degree of structural diversity; for instance, in Rb- and Cs-PolyP (a) and Li- and K-PolyP (b), tetrahedra form infinite two-membered chains with a repeating structural unit consisting of two tetrahedra. The anionic chains of Na-PolyP and Maddrell high-temperature salt (c) are three-membered, while Pb- and Ca-PolyP (d) are four-membered. In Ag-PolyP and Na-Kurrol A salt (e), as well as Na-Kurrol B salt (f), the helices in the chains are twisted differently [20] (Figure 1).

Furthermore, the stability and degradation rate of linear PolyP in aqueous solutions are dynamically governed by this chain length and local environmental factors. While relatively stable at neutral pH and 25 °C, PolyP degrades more rapidly at higher temperatures or altered pH levels. This degradation occurs through two distinct, uncoupled pathways: a zero-order hydrolytic removal of terminal monophosphates that accelerates between pH 7 and 10 and a first-order internal rearrangement forming cyclic metaphosphates, which is heavily favored under acidic conditions (pH 3–5). The intrinsic degradation rate increases with chain length up to approximately ten units before plateauing for chains exceeding 100 units [21]. Therefore, long-chain PolyP tends to form more stable nanoparticles, whereas medium- and short-chain PolyP are more prone to forming bioactive condensates [22].

Ultimately, these structural features—charge density, chain-length-dependent condensation, and responsive degradation kinetics—not only determine the distribution and stability of PolyP within and outside cells but also provide the molecular basis for its participation in biological processes such as energy metabolism and mineralization regulation [23].

### 2.2. Biosynthesis and Metabolism in Organisms

PolyP naturally exists in mammalian platelets, osteoblasts, and body fluids, playing a significant role in bone metabolism [24,25]. The chain length of naturally derived PolyP varies markedly: in microorganisms, it often comprises long chains of hundreds to thousands of phosphate units, whereas in mammals, PolyP chain length typically ranges from 60 to 100 phosphate units [26]. In bacteria, PolyP synthesis uses ATP as a substrate and is catalyzed by polyphospho kinase (PPK) enzymes, and it is mainly stored in PolyP granules or acidocalcisomes [27]. In mammalian cells, platelets are the main cells in the body that store PolyP, with a concentration of up to 130 mM (as phosphate). PolyP is packaged in dense granules and released extracellularly after platelet activation [28]. After release, PolyP can be hydrolyzed stepwise from the terminal end by exophosphatases such as alkaline phosphatase (ALP), releasing orthophosphate and generating energy [29]. This hydrolysis process provides phosphate substrates and metabolic energy for bone mineralization.

## 3. Mechanisms of PolyP in Promoting Osteogenesis

Osteogenesis requires coordinated cell differentiation, extracellular matrix mineralization, and energy metabolism [30,31]. PolyP uniquely integrates these processes through multiple complementary mechanisms. These aspects are summarized in Table 1 and will be discussed below.

### 3.1. Participation in Bone Mineralization as a Phosphate Donor

Bone mineralization centers on HA formation [32], requiring continuous phosphate and calcium ion (Ca^2+^) supplies [33]. PolyP serves as an effective alternative to traditional β-glycerophosphate by acting as a concentrated phosphate reservoir [34]. During mineralization, PolyP is hydrolyzed by alkaline phosphatase (ALP) to release inorganic phosphate (Pi), which couples with Ca^2+^ to form amorphous calcium phosphate and eventually crystalline HA [35]. PolyP nanoparticles can achieve mineralization outcomes comparable to β-glycerophosphate in human bone marrow mesenchymal stem cells (hBMSCs) [36] while simultaneously regulating intracellular calcium concentrations to activate osteogenesis-related signaling pathways [37].

### 3.2. Participation in Energy Metabolism as an Energy-Storing Molecule

**Figure 2:**
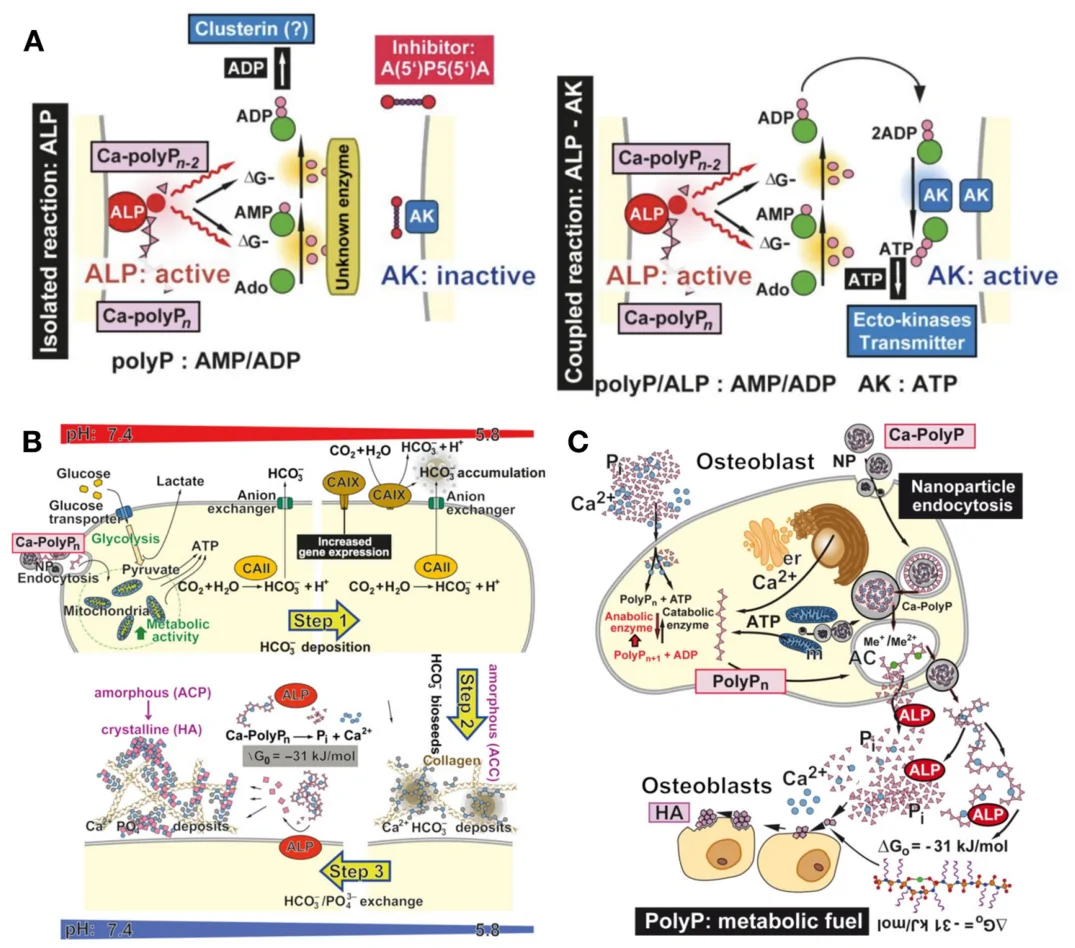
The role of PolyP in regulating cellular energy metabolism and driving biomineralization. This figure illustrates how PolyP serves as an energy-storing molecule to fuel extracellular bone mineralization under both physiological and hypoxic conditions. (**A**) Mechanistic pathways demonstrating the PolyP-driven extracellular enzymatic synthesis of adenosine diphosphate (ADP) and adenosine triphosphate (ATP), highlighting PolyP’s function as a high-energy phosphate donor (Reproduced with permission from Ref. [38]. Copyright **2017** The Company of Biologists Ltd.). (**B**) A proposed model of PolyP-mediated hydroxyapatite (HA) formation on bone cells under hypoxic conditions, where PolyP acts as a metabolic fuel to maintain cellular activity and promote continuous HCO_3_^−^/PO_4_^3−^ exchange for mineralization. (Reproduced with permission from Ref. [44]. Copyright 2015 John Wiley and Sons). (**C**) A schematic outline illustrating the cellular endocytosis of PolyP nanoparticles, subsequent intracellular ATP generation, and the comprehensive biomineralization process resulting in extracellular HA deposition onto bone cells (Reproduced with permission from Ref. [43]. Copyright **2015** The Company of Biologists Ltd.).

Beyond phosphate donation, PolyP contains high-energy phosphoanhydride bonds that actively participate in mineralization energy metabolism [38] (Figure 2A). Upon hydrolysis, these bonds release free energy (~30.5 kJ/mol per unit) comparable to ATP, allowing a 40-unit chain to store substantially more energy than a single ATP molecule [13]. Crucially, extracellular hydrolysis by ALP generates adenosine diphosphate (ADP) and ATP, uniquely fueling the extracellular bone mineralization process—an advantage unmatched by traditional bone repair materials [39,40].

Furthermore, PolyP modulates energy metabolism by acting as a structural component and activator of the mitochondrial permeability transition pore (mPTP) [41], inducing mitochondrial elongation in osteoblasts [42]. This metabolic response is highly context-dependent: in MC3T3-E1 cells, PolyP-mediated mPTP opening alters the ADP/ATP ratio as an early trigger for cellular calcification [42], whereas in SaOS-2 cells, amorphous calcium polyphosphate nanoparticles (CPP-NP) induce ALP translocation and significantly increase intracellular ATP levels [43] (Figure 2C). Thus, PolyP acts as a dynamic osteogenic regulator via mPTP and ATP modulation. Moreover, under hypoxia, PolyP functions as a metabolic fuel to sustain mineralization, upregulating hypoxia-inducible factor 1 alpha (HIF-1α) and carbonic anhydrase IX to energetically support bone formation [44] (Figure 2B).

### 3.3. Regulation of Osteogenesis-Related Signaling Pathways

PolyP actively regulates intracellular signaling networks to drive osteogenic differentiation. It activates the Wnt/β-catenin pathway [45], upregulating β-catenin, cyclin D1, and osteogenic markers such as Runt-related transcription factor 2 (Runx2), osteocalcin (OCN) and collagen type I (COL-1) in hBMSC [46,47]. Additionally, PolyP stabilizes fibroblast growth factor (FGF)-2 to prolong receptor binding and activate extracellular signal-regulated kinase (ERK) 1/2 phosphorylation, thereby upregulating matrix metalloproteinase 1 (MMP-1), osteopontin (OPN) and osteoprotegerin (OPG) [48]. This ERK activation via basic fibroblast growth factor receptor (bFGFR) is rapid and specific [49]. PolyP nanoparticles also upregulate SRY-box transcription factor 9 (Sox9) and Runx2 while downregulating cathepsin-K, demonstrating strong bone anabolic activity [50]. Furthermore, PolyP facilitates correct collagen folding by binding to cyclophilin B (CypB) and inhibiting its peptidyl-prolyl cis-trans isomerase (PPIase) activity [51]. Crucially, PolyP cross-talks with the AMP-activated protein kinase (AMPK) pathway—a core regulator of cellular energy—by modulating free phosphate homeostasis and regulating xenotropic and polytropic retrovirus receptor 1 (XPR1) and inositol hexakisphosphate kinase 1/2 (IP6K1/2), effectively coupling mitochondrial energy metabolism with osteogenesis [52].

**Figure 3:**
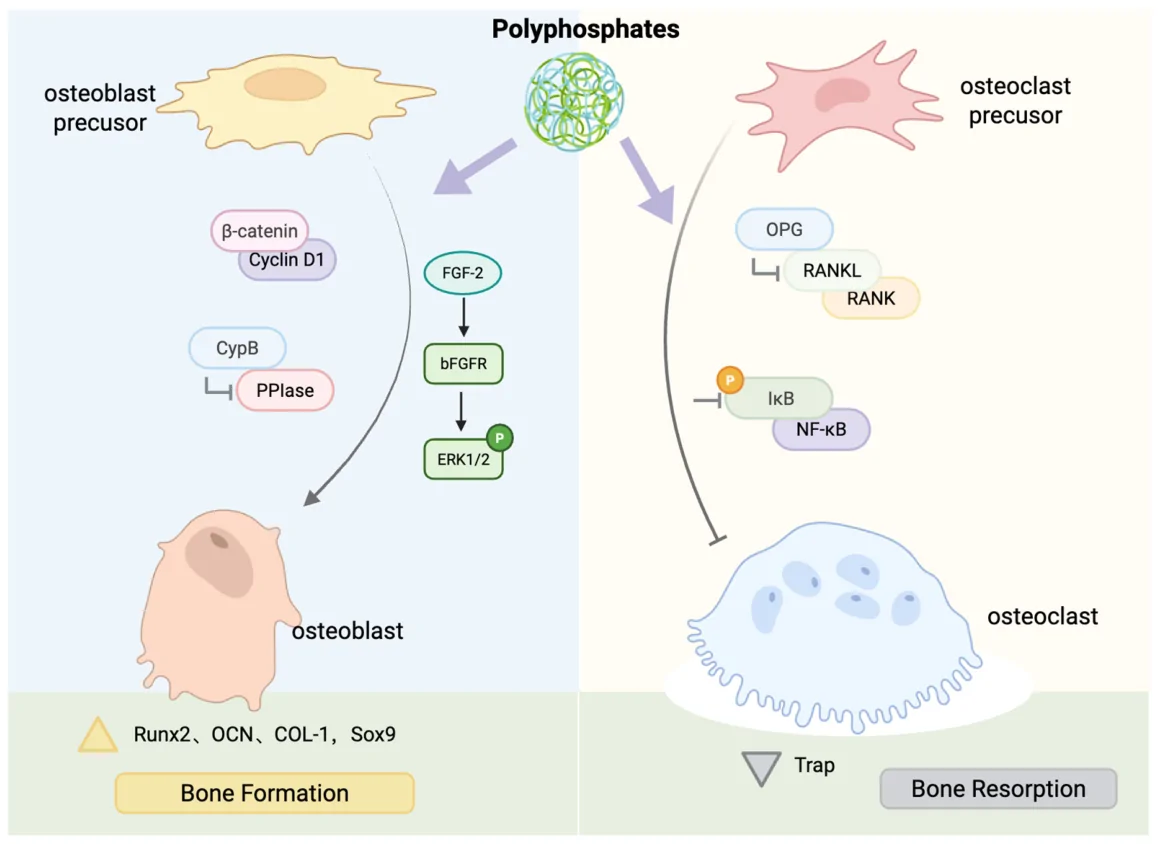
The regulation of osteogenesis-related signaling pathways. This schematic illustrates the dual regulatory mechanisms of PolyP on osteoblast-mediated bone formation and osteoclast-mediated bone resorption. The left panel depicts the stimulatory effects of PolyP on osteogenic lineage cells. PolyP promotes bone formation by activating the Wnt/β-catenin and fibroblast growth factor/extracellular signal-regulated kinase (FGF/ERK) signaling pathways and by inhibiting cyclophilin B/peptidyl-prolyl cis-trans isomerase (CypB/PPIase) to facilitate correct collagen folding, which synergistically upregulate key osteogenic markers. Conversely, the right panel illustrates the inhibitory effects of PolyP on osteoclastogenesis. PolyP mitigates bone resorption by upregulating osteoprotegerin (OPG) to neutralize receptor activator of nuclear factor κB ligand (RANKL), blocking the nuclear factor kappa B (NF-κB) signaling pathway, and directly suppressing tartrate-resistant acid phosphatase (TRAP) enzyme activity. Together, these coordinated regulations demonstrate how PolyP shifts the bone homeostatic balance toward regeneration. (Created in BioRender, https://BioRender.com/zaihznb).

Beyond osteoblasts, bone homeostasis requires a balance with osteoclast functions [53], heavily regulated by the OPG/receptor activator of nuclear factor κB ligand (RANKL) system [54]. OPG binds to RANKL, neutralizing its function [55]. PolyP exhibits dual anabolic activity by inducing bone morphogenetic protein 2 (BMP-2) expression in osteoblasts while simultaneously inhibiting osteoclast maturation [56]. Mechanistically, this is achieved by upregulating OPG to neutralize RANKL activity [57], inhibiting inhibitor of nuclear factor kappa B alpha (IκBα) phosphorylation to block the nuclear factor kappa B (NF-κB) signaling pathway [58], and directly suppressing tartrate-resistant acid phosphatase (TRAP) activity, particularly with long-chain PolyP (≥300 units) variants [59]. In summary, PolyP regulates both osteoblast and osteoclast activities to construct an immune–bone coupling microenvironment favorable for regeneration (Figure 3).

### 3.4. Other Mechanisms

PolyP further enhances bone regeneration through indirect means, notably angiogenesis and antibacterial activity. Because bone regeneration is intimately coupled with angiogenesis [60], ATP derived from PolyP hydrolysis promotes human umbilical vein endothelial cell (HUVEC) proliferation and tube formation [61]. Furthermore, PolyP matrices stimulate osteoblasts to secrete angiogenic factors like vascular endothelial growth factor (VEGF) and basic fibroblast growth factor (bFGF) [62,63]. Additionally, PolyP exhibits significant antibacterial properties [64], encapsulating and killing bacteria while simultaneously fueling wound healing with metabolic energy [65], offering distinct advantages for treating infected bone defects [66].

**Table 1:** Mechanisms of PolyP in promoting osteogenesis.

Domain	Mechanism	References	Detailed Mechanism and Effects
Bone Mineralization	Phosphate Donor	[33,34,35,36]	Serves as phosphate storage; hydrolyzed by alkaline phosphatase (ALP) to release inorganic phosphate (Pi), forming amorphous calcium phosphate and eventually hydroxyapatite (HA).
	Calcium Ion Regulation	[33,34,35,36,37]	Regulates intracellular calcium ion concentration. Activates multiple osteogenic signaling pathways by inducing an increase in intracellular calcium ions.
Energy Metabolism	Metabolic Fuel	[13,38,39,40]	High-energy phosphoanhydride bonds generate adenosine diphosphate (ADP) /adenosine triphosphate (ATP) upon hydrolysis, directly fueling extracellular bone mineralization.
	Mitochondrial Regulation	[41,42,43,44]	Activates components of the mitochondrial permeability transition pore (mPTP); induces mitochondrial elongation/swelling; maintains growth/mineralization under hypoxia via hypoxia-inducible factor 1 alpha (HIF-1α) and carbonic anhydrase IX upregulation.
Cell Signaling	Wnt/β-catenin Pathway	[45,46,47]	Activates the signaling pathway to upregulate β-catenin and cyclin D1, promoting osteogenic markers.
	FGF/ERK Pathway	[48,49]	Stabilizes fibroblast growth factor (FGF)-2 for receptor binding, activating extracellular signal-regulated kinase (ERK)1/2 phosphorylation to upregulate osteogenic genes.
	Protein Folding	[51]	Binds to cyclophilin B (CypB), inhibiting peptidyl-prolyl cis-trans isomerase (PPIase) activity to regulate collagen folding.
	AMPK Pathway	[52]	Regulates intracellular free phosphate homeostasis, impacting phosphate transporter xenotropic and polytropic retrovirus receptor 1 (XPR1) and inositol hexakisphosphate kinase (IP6K) 1/2, thereby modulating AMP-activated protein kinase (AMPK) phosphorylation.
Cell Homeostasis	Osteoclast Differentiation	[56,57,58]	Upregulates osteoprotegerin (OPG) expression to neutralize receptor activator of nuclear factor κB ligand (RANKL); inhibits inhibitor of nuclear factor kappa B alpha (IκBα) phosphorylation to block NF-κB signaling.
	Bone Resorption	[59]	Long-chain PolyP (≥300 units) directly inhibits tartrate-resistant acid phosphatase (TRAP) activity, reducing the resorption pit area.
Indirect Mechanisms	Angiogenesis	[61,62,63]	ATP produced by PolyP hydrolysis promotes human umbilical vein endothelial cell (HUVEC) tube formation; stimulates vascular endothelial growth factor (VEGF) and basic fibroblast growth factor (bFGF) secretion to couple angiogenesis.
	Antibacterial Activity	[64,65,66]	Encapsulates and kills bacteria during wound healing while providing metabolic energy for tissue repair.

## 4. Design and Applications of PolyP in Biomaterials

**Figure 4:**
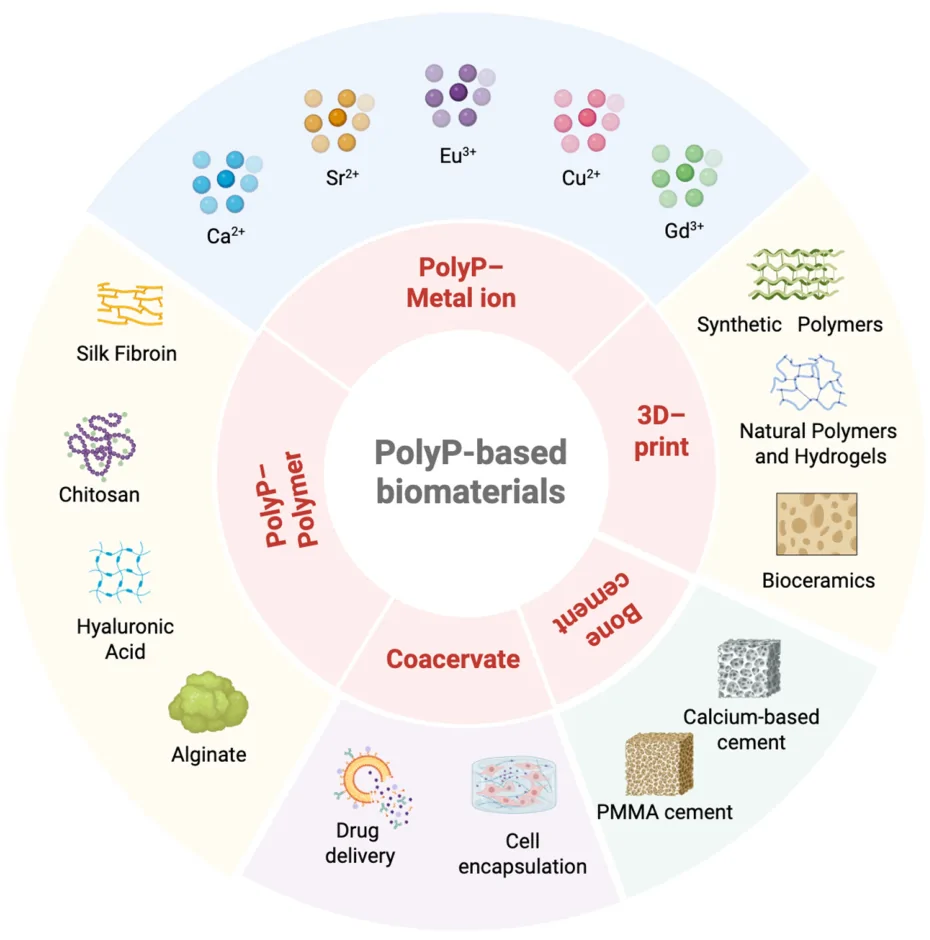
Examples of the use of PolyP in biomaterials for bone tissue engineering. This schematic illustrates the diverse components and final forms of PolyP-incorporated material systems. The presented examples are classified into five main categories according to their composition and design rationale: (1) PolyP–metal ion and (2) PolyP–polymer represent fundamental PolyP-based materials formed by compounding PolyP with specific osteogenic metal ions (e.g., Ca^2+^, Sr^2+^) and various polymers (e.g., silk fibroin, chitosan). (3) Coacervates refer to the PolyP-based materials formed by liquid–liquid phase separation in the presence of cationic polyelectrolytes or metal cations. Unlike solid precipitates, coacervates remain highly viscous yet retain substantial water and fluidity and are assembled via reversible noncovalent interactions. (4) 3D-printed scaffolds and (5) bone cements represent PolyP-modified materials, in which PolyP is doped into traditional 3D printing matrices or cement frameworks to modify and enhance their physicochemical and biological properties. (Created in BioRender, https://BioRender.com/hmn3nd5).

The design of PolyP-incorporated biomaterials is fundamentally driven by the structural heterogeneity, degradation kinetics, and physicochemical properties of PolyP. Because branched PolyP is highly unstable in aqueous environments and cyclic PolyP lacks defined biological functions, biomaterial design predominantly utilizes linear PolyP. The core design principles rely on the robust capacity of PolyP to interact with various matrix materials. Crucially, the mode of binding to substrate materials influences the degradation characteristics and subsequent biological activity of PolyP: noncovalently bound PolyP (e.g., through physical blending or electrostatic interactions) is readily released, whereas covalently bound PolyP can exert a more sustained therapeutic effect. By translating these unique physicochemical and degradative characteristics into targeted design strategies, various forms of PolyP-involved bone repair materials have been rationally developed to provide tunable degradation and sustained therapeutic efficacy. According to the structural design and form of application, they can be categorized into metal ion–PolyP complexes, PolyP–polymer composite materials, bone cements, 3D printed/electrospun scaffolds and coacervate materials (Figure 4). The specific structure, design strategies, applications and binding modes of these biomaterials are summarized in Table 2.

### 4.1. Metal Ion–PolyP Complexes

The primary design principle for these complexes utilizes the electrostatic properties of PolyP. PolyP is composed of phosphate units linked by high-energy phosphoanhydride bonds. At neutral pH, monohydrogen phosphate units carry a monovalent negative charge and can bind with cations to form salts [16]. Calcium polyphosphate (CPP) was the earliest PolyP-based bone repair material to be studied. Amorphous CPP-NP and calcium polyphosphate microparticles (CPP-MP) slowly release phosphate and calcium ions under physiological conditions, providing cellular energy and demonstrating superior bone regeneration compared to traditional beta-tricalcium phosphate (β-TCP) control groups in critical-size calvarial defects [67]. Combining CPP with amorphous calcium carbonate (ACC) further enhances osteogenic effects through the complementary induction of ALP and carbonic anhydrase, where PolyP specifically stabilizes the amorphous phase of ACC and regulates its crystallization process within an ALP-mediated hydration environment [68,69,70].

Substituting calcium with other osteogenic metal ions provides additional functionalization. Strontium ions possess the dual functions of promoting osteogenesis and inhibiting bone resorption. Amorphous strontium PolyP microparticles accelerate calvarial defect healing by significantly upregulating BMP-2 and ALP while exerting a weaker upregulation effect on sclerostin (SOST) compared to CPP [71]. Strontium-doped calcium polyphosphate (SCPP) combined with autologous bone marrow mesenchymal stem cells (BMSCs) is also highly effective for the treatment of femoral head osteonecrosis [72]. Furthermore, trace element doping introduces targeted bioactivities: 5% europium-doped calcium polyphosphate (EuCPP) porous scaffolds promote BMSC migration, enhance ALP/OPN secretion, and increase the OPG/RANKL ratio [73]; 0.05% copper-doped calcium polyphosphate (CCPP)/dopamine (DOPA)/Collagen scaffolds using DOPA-linked collagen stabilize HIF-1α to significantly upregulate VEGF alongside osteogenic genes such as ALP, OCN, and OPN [74]; and Gd^3+^ complexation for preparing PolyP-Gd rapidly promotes HA formation in SaOS-2 cells while upregulating BMP-2 and CoL-1 expression [75]. Collectively, these ionic modifications provide multi-dimensional osteogenic properties.

### 4.2. PolyP–Polymer Composite Materials

**Figure 5:**
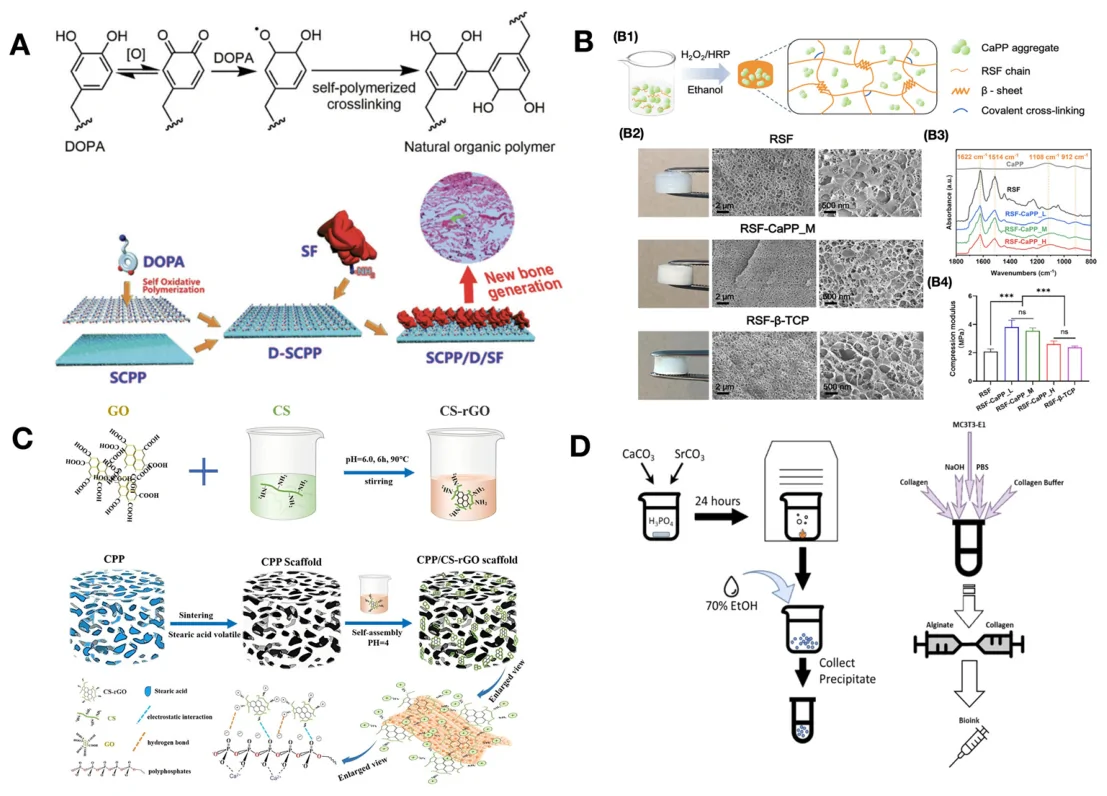
Formulation strategies for PolyP–polymer composite materials. This figure shows how PolyP can be integrated with diverse natural and synthetic polymers to construct bioactive scaffolds and hydrogels with enhanced mechanical and osteogenic properties. (**A**) The mechanism of the two-step surface modification of the SCPP porous scaffold, utilizing DOPA as a linker to immobilize SF for accelerated bone regeneration (Reprinted with permission from Ref. [76]. Copyright **2016** Royal Society of Chemistry). (**B**) The design strategy (**B1**), structural characterization (**B2**), ATR-FTIR spectra (**B3**) and compressive modulus properties (**B4**) of RSF hydrogels combined with varying degrees of CPP, demonstrating tunable network formation, *** *p* < 0.001 (Reprinted with permission from Ref. [77] Copyright **2025** Royal Society of Chemistry). (**C**) The fabrication process and formation mechanism of a self-assembled CS-rGO coating on porous CPP scaffolds, primarily driven by electrostatic interactions and hydrogen bonding. (Reproduced from Ref. [78]. Copyright **2022** IOP Publishing). (**D**) A schematic diagram outlining the preparation and bioprinting process of SCPP-doped alginate/collagen double-network hydrogels for cell encapsulation (Reprinted from Ref. [82]).

The design strategy for composite materials involves combining the bioactivity of PolyP with the structural and mechanical scaffolding provided by natural or synthetic polymers. Various natural polymers, including silk fibroin (SF), chitosan (CS), hyaluronic acid (HyA), alginate, and gelatin, are utilized as the foundational framework in combination with PolyP to construct bone repair scaffolds. Using a polydopamine coating to immobilize SF onto SCPP produces SCPP/DOPA/SF composite scaffolds with enhanced compressive strength, improved HUVEC and MG63 cell proliferation, and augmented VEGF and bFGF secretion, significantly accelerating new bone formation in rabbit calvarial defect models [76] (Figure 5A). In regenerated silk fibroin (RSF) hydrogels fabricated via enzymatic crosslinking and ethanol treatment, the PolyP degree of polymerization modulates Ca^2+^ release, with a polymerization degree of 22 yielding the optimal osteogenic differentiation of BMSCs in vitro [77] (Figure 5B). Similarly, applying a self-assembled chitosan-grafted reduced graphene oxide (CS-rGO) coating onto porous CPP scaffolds, or combining CPP with chitosan and pigeonite, significantly improves biomineralization, increases the OPG/RANKL ratio to mitigate aseptic loosening, and promotes bone formation in rat tibial defect models [78,79] (Figure 5C). For hydrogel systems, PolyP can be covalently grafted onto HyA to construct HAX-PolyP hydrogels that provide sustained, immobilized osteogenic stimulation, upregulating Runx2, ALP, and OCN in MC3T3-E1 cells [80]. Furthermore, incorporating CPP or SCPP into alginate and alginate/collagen double-network matrices allows for optimal cell encapsulation (effective even at low 0.3–0.5 mg/mL SCPP doses), ensuring high porosity and supporting early mineralization [81,82] (Figure 5D). Finally, modifying gelatin with PolyP enhances biomimetic mineralization by increasing in situ mineralization saturation from 25% to more than 40%, boosting the compressive strength and elastic modulus of the composite by 57.93% and 132.55%, respectively, thereby promoting osteogenesis [83].

### 4.3. PolyP-Incorporated Bone Cements

Bone cements play a crucial role in clinical applications such as vertebroplasty and joint replacement due to their injectability, self-setting capabilities, and favorable mechanical properties for filling defects [84,85]. Building upon these advantages, the incorporation of PolyP aims to further optimize setting times, tailor mechanical performance, and enhance the overall bioactivity of the matrices. Polymethyl methacrylate (PMMA) bone cement is a commonly used material in clinical bone repair [86], yet it lacks sufficient bioactivity. Incorporating polydopamine-coated SCPP particles into PMMA cements to form polydopamine/SCPP/PMMA not only promotes MG63 cell proliferation, ALP activity, and BMP-2/VEGF secretion but importantly reduces the potentially damaging curing reaction temperature [87]. Additionally, integrating sodium polyphosphate nanoparticles (NPP-NP) or CPP-NP into PMMA modulates surface hardness and elasticity, providing the cement with remarkable self-healing properties by strongly accelerating the closure of microcracks through the deposition of newly formed mineralic material [70]. Beyond PMMA, advanced calcium-based systems have been engineered. Calcium polyphosphate bone cement (CPPC) offers composition-controlled setting times and effectively induces MG63 and ST2 cell mineralization, promoting new bone formation in rat skull defect models better than traditional calcium phosphate cements [88]. Similarly, tricalcium silicate/calcium polyphosphate/polyvinyl alcohol (C_3_S/CPP/PVA) composite cements achieve highly tunable setting times (5.5–37.5 min) and robust compressive strengths (~28 MPa), promoting MC3T3 cell proliferation without observed cytotoxicity [89].

### 4.4. 3D-Printed and Electrospun Scaffolds

3D printing enables the customization of personalized scaffolds with precise external geometry and internal pore architecture based on patient imaging data [90,91], while electrospinning excels at mimicking the nanofibrous network of the native extracellular matrix [92,93]. The combination of these advanced manufacturing techniques with PolyP-incorporated materials opens new avenues for constructing functionalized bone repair scaffolds. In 3D printing, encapsulating CPP nanoparticles within polycaprolactone (PCL) supports SaOS-2 cell growth and upregulates chemokines such as stromal cell-derived factor 1 (SDF-1) [94,95]. When coprinted with β-TCP, PolyP forms a molten glass binder during heat treatment that embeds CPP-NP and β-TCP particles, yielding a scaffold with ~73% porosity that effectively releases PolyP to promote BMSC mineralization [96]. PolyP also enhances the mechanical properties of alginate/poly(ethylene glycol) diacrylate dual-network hydrogels through the synergy of covalent and ionic crosslinking networks, boosting cellular ATP content and migration [97]. Furthermore, using polyvinyl alcohol as a binder for the solid freeform fabrication of CPP powder creates structures with 35 vol.% pore content and 33.86 MPa compressive strength for in vitro cartilage formation [98], while printing CPP with alginoplast forms porous, cauliflower-shaped carbonate apatite microstructures ideal for cell attachment [99].

Electrospinning incorporates PolyP into PCL/poly(L-lactic acid) (PLLA) nanofibers, promoting the osteogenic differentiation of induced pluripotent stem cells (iPSCs) [47]. Additionally, coating electrospun poly(ε-caprolactone) meshes with amorphous PolyP nanoparticle-loaded collagen creates active barrier membranes with increased flexibility and stretchability that promote human bone marrow mesenchymal stem cell (hBMSC) metabolic activity [100]. Electrospun PCL/Sr-PolyP mats functionalized with ascorbyl palmitate can induce calcium phosphate deposition in simulated body fluid (SBF) while releasing ascorbic acid [101], and SCPP scaffolds can be designed for the controlled, dual-drug release of tetracycline and matrix metalloproteinase-2 to repair rabbit skull defects [102].

### 4.5. Coacervate Materials

Coacervate is a macromolecule-rich aqueous liquid phase generated via liquid–liquid phase separation (LLPS). Because PolyP is negatively charged at neutral and alkaline pH, it undergoes LLPS in the presence of cationic polyelectrolytes or metal cations. This process is concentration-dependent: at low metal ion concentrations, cations occupy “cage-like” binding sites within the PolyP chain; at high concentrations, external binding leads to inter-chain crosslinking and phase separation [103]. Unlike solid precipitates, these condensates remain highly viscous yet retain substantial water and fluidity, assembled via reversible noncovalent interactions. Notably, the formation of these aggregates is a key step for PolyP to exert its biological activity, whereas nanoparticles primarily serve as a storage form [104]. PolyP coacervates excel as an encapsulation matrix for BMSCs, supporting cell survival, proliferation, and differentiation within the condensate [104], and serve as advanced wound dressings providing both ATP to fuel healing and antibacterial effects [105]. Exploiting their reversible phase transitions, sophisticated core–shell drug delivery systems have been developed—such as a dexamethasone (Dex)-loaded CPP-NP core surrounded by an outer shell of ascorbic acid (AA)-loaded aggregates—enabling distinct release kinetics for combination therapy in bone tissue engineering [106].

**Table 2:** Design and application of PolyP-incorporated biomaterials.

Material Type		Structure	Application	Binding Mode
Metal ion composites	Ca	~40 units	Amorphous calcium polyphosphate microparticles/nanoparticles (CPP-MP/NP): Slowly release Pi/Ca^2+^ and provide cellular energy [67].	Ionic chelation
		~40 units	Calcium polyphosphate (CPP) and CPP/amorphous calcium carbonate (ACC) particles: Promote bone regeneration; synergistic effects with ACC [68,69].	Ionic chelation
		~40 units	PolyP/ACC nanoparticles: Stabilize ACC for synergistic bone repair [70].	Physical blending and encapsulation
	Sr	~40 units	Amorphous Sr-PolyP microparticles: Promote bone marrow mesenchymal stem cell (BMSC) mineralization and accelerate in vivo bone healing [71].	Ionic substitution and doping
		NA	Strontium-doped calcium polyphosphate (SCPP) /BMSC composite: Treats femoral head necrosis [72].	Ionic substitution and doping
	Eu	NA	Europium-doped calcium polyphosphate (EuCPP) porous scaffold: Promotes BMSC migration and in vivo bone regeneration [73].	Ionic substitution and doping
	Cu	NA	Calcium polyphosphate bone cement (CCPP)/dopamine (DOPA)/collagen scaffold: Upregulates osteogenic genes for new bone formation [74].	Covalent and chemical bonding
	Gd	~40 units	PolyP-Gd complex: Promotes hydroxyapatite (HA) formation and upregulates osteogenic genes [75].	Ionic bonding and crosslinking
Polymer composites	Silk fibroin	NA	SCPP/DOPA/silk fibroin (SF) scaffold: Promotes angiogenesis and accelerates in vivo bone formation [76].	Electrostatic and hydrogen bonding; covalent and chemical bonding
		Linear; 8/22/121 units	Regenerated silk fibroin (RSF)-CPP hydrogel: Promotes in vitro BMSC osteogenic differentiation [77].	Electrostatic and hydrogen bonding
	Chitosan	Linear	CPP/chitosan-reduced graphene oxide (CS-rGO) scaffold: Enhances BMSC biomineralization and osteogenic differentiation [78].	Electrostatic and hydrogen bonding
		Linear	CS/CPP/pigeonite scaffold: Promotes BMSC differentiation and in vivo bone formation [79].	Physical adsorption and adhesion
	Hyaluronic acid	13 units	HAX-PolyP hydrogel: Provides sustained osteogenic stimulation [80].	Covalent and chemical bonding
	Alginate	NA	Alginate/CPP double-network hydrogel: Supports cell proliferation and osteogenic differentiation [81].	Physical blending and encapsulation
		NA	SCPP-crosslinked alginate/collagen hydrogel: Promotes cell survival and early mineralization [82].	Ionic bonding and crosslinking
	Gelatin	NA	PolyP-modified gelatin: Improves compressive strength and osteogenic induction [83].	Covalent and chemical bonding
Bone cement	PMMA cement	NA	Polydopamine/SCPP/poly(methyl methacrylate) (PMMA) composite cement: Promotes osteoblast proliferation [87].	Physical adsorption and adhesion
		~40 units	Sodium polyphosphate nanoparticles (NPP-NP) or calcium polyphosphate nanoparticles (CPP-NP) in PMMA: Modulates mechanics and improves self-healing properties [70].	Physical blending and encapsulation
	Calcium-based cement	Linear	CPPC: Induces in vitro mineralization and in vivo bone formation [88].	Covalent and chemical bonding
		Linear; ~40 units	Tricalcium silicate/calcium polyphosphate/polyvinyl alcohol (C3S/CPP/PVA) composite cement: Promotes osteoblast proliferation and osteogenic differentiation [89].	Physical adsorption and adhesion
3D-printed and electrospun materials	Synthetic polymers	~40 units	CPP-NP/polycaprolactone (PCL) three-dimensional (3D)-printed scaffold: Supports cell growth and upregulates chemokines [94].	Physical blending and encapsulation
		NA	PolyP-incorporated PCL/poly(L-lactic acid) (PLLA) nanofibers: Promotes induced pluripotent stem cell (iPSC) osteogenic differentiation [47].	Physical blending and encapsulation
		~40 units	Amorphous CPP/collagen-coated PCL mat: Promotes BMSC attachment and angiogenesis [100].	Physical blending and encapsulation
		~40 units	Sr-PolyP/ascorbyl palmitate/PCL fibers: Improves bioactivity and cell compatibility [101].	Physical blending and encapsulation
	Bioceramics	~40 units	PolyP/beta-tricalcium phosphate (β-TCP) 3D-printed scaffold: Releases PolyP to promote BMSC proliferation and mineralization [96].	Physical blending and encapsulation
		Linear	Powder-printed CPP: Supports in vitro cartilage formation for osteochondral repair [98].	Physical blending and encapsulation
		~40 units	SCPP scaffold with tetracycline/matrix metalloproteinase (MMP)-2: Dual-drug release for promoting in vivo bone regeneration [102].	Physical blending and encapsulation
	Natural polymers and hydrogels	Linear	Alginate/poly(ethylene glycol) diacrylate/PolyP double-network hydrogel: Boosts cellular adenosine triphosphate (ATP), migration, and osteogenesis [97].	Ionic chelating
		Linear	Printable CPP/alginoplast powder: Forms porous carbonate apatite for cell attachment [99].	Physical blending and encapsulation
Coacervate-related	Cell encapsulation	Linear; ~40 units	PolyP coacervate matrix: Supports BMSC survival, proliferation, and differentiation [104].	Ionic bonding and crosslinking
	Drug delivery	~40 units	Dexamethasone (Dex)-CPP/ascorbate–coacervate core–shell system: Enables multikinetic drug delivery [106].	Ionic bonding and crosslinking

## 5. Clinical Translation Studies

### 5.1. Preclinical Animal Studies

Numerous animal studies have confirmed the bone repair efficacy of PolyP-incorporated materials. Wang et al. demonstrated in a rabbit femoral defect model that lithium-doped CPP composite scaffolds promoted bone regeneration, a mechanism associated with the activation of the Wnt signaling pathway [107]. Kang et al. confirmed in a rabbit steroid-induced femoral head necrosis model that SCPP combined with autologous bone marrow mononuclear cells promoted bone repair and upregulated VEGF expression [72]. These studies lay the foundation for the clinical translation of PolyP-incorporated materials. Comeau et al. determined the ability of CPP to support cell proliferation and differentiation in vivo. Along with CPP, morselized cancellous bone and HA/tricalcium phosphate particles were seeded with bone marrow stromal cells and implanted subcutaneously in the backs of mice. CPP was superior to HA/tricalcium phosphate and morselized bone in its ability to support cell survival in vivo and has high potential as a graft material for bone regeneration [108].

Pilliar et al. tested two types of CPP particles prepared under different annealing conditions, producing either slower- or faster-degrading particles. These CPP particles, implanted into rabbit and sheep femoral condyles, demonstrated rapid bone ingrowth without a chronic inflammatory or cytotoxic response. Based on bone ingrowth characteristics, the more rapidly degrading CPP was deemed desirable [109].

Addressing the need in orthopedic surgery for materials that combine hemostasis and osteogenesis, Zhu et al. developed a wax-like codoped PolyP by chelating Na-PolyP with cationic ions (Ca^2+^, Mg^2+^, and Sr^2+^). The resulting material was found to be ductile and degraded optimally, releasing Mg^2+^ and Sr^2+^ ions, significantly increasing the expression of osteogenesis-related genes and bone-related proteins. The material exhibits excellent hemostatic and osteogenic properties, making it promising for bone tissue restoration [110].

### 5.2. Clinical Trials

The safety of PolyP has been preliminarily validated. Alkaabi et al. conducted the first clinical trial of PolyP for human alveolar bone defect repair [111,112]. This trial enrolled eight patients with alveolar clefts, who received CPP-MP either alone or in combination with biphasic calcium phosphate (BCP). The results showed that no local or systemic adverse reactions occurred in any patient, confirming the safety of PolyP. However, the group treated with CPP alone exhibited suboptimal bone filling due to insufficient material stability, whereas the group receiving the combination with BCP demonstrated better bone healing outcomes. This finding suggests that PolyP needs to be combined with a suitable carrier material to achieve optimal repair efficacy.

Currently, this remains the only one published human clinical trials investigating PolyP for bone regeneration. The lack of long-term in vivo degradation and systemic toxicity data from standardized large animal models may be one of the reasons limiting the conducting of clinical trials, as regulatory agencies require these data before approving broader human trials.

## 6. Challenges and Outlook

While recent advancements have established PolyP as a multi-functional agent that couples metabolic energy supply with structural support in bone tissue engineering, several critical bottlenecks must be overcome to realize its full clinical potential. First, the precise relationship between PolyP chain length and osteoinductive activity remains ambiguous. Future research must determine the optimal chain lengths tailored for specific applications to maximize bioactivity while preventing adverse coagulation or inflammatory responses. Furthermore, current PolyP-incorporated materials often exhibit degradation rates that are misaligned with new bone maturation. Advancing intelligent, responsive materials—such as multi-layer core–shell structures or specifically crosslinked coacervates—will be essential to achieve synchronized release kinetics. Additionally, because bone regeneration relies on complex signaling networks, future designs should prioritize the synergistic delivery of PolyP with specific growth factors (e.g., BMP-2, VEGF) or small-molecule drugs to exploit PolyP’s unique metabolic fueling capabilities.

Beyond material design, a rigorous and structured pathway for clinical translation is imperative. Existing clinical data are largely limited to small-scale safety validations. The transition from bench to bedside requires the establishment of Good Manufacturing Practice-compliant synthesis protocols to ensure batch-to-batch consistency. Subsequent in vivo evaluations must utilize standardized large animal models to provide comprehensive data on long-term biomechanical stability and systemic pharmacokinetics. Ultimately, well-designed, multi-center randomized controlled trials are needed to compare specific PolyP formulations against current clinical gold standards for defined orthopedic indications. By addressing these translational gaps, next-generation PolyP biomaterials can evolve from passive bone substitutes into active regulators of the metabolic and immune microenvironments, establishing a new paradigm in regenerative nanomedicine.

## Data Availability

No new data were created or analyzed in this study. Data sharing is not applicable to this article.

## Abbreviations

The following abbreviations are used in this manuscript:

AA	ascorbic acid
ACC	amorphous calcium carbonate
ADP	adenosine diphosphate
ALP	alkaline phosphatase
AMPK	AMP-activated protein kinase
ATP	adenosine triphosphate
BCP	biphasic calcium phosphate
β-TCP	beta-tricalcium phosphate
bFGF	basic fibroblast growth factor
bFGFR	basic fibroblast growth factor receptor
BMP-2	bone morphogenetic protein 2
BMSC	bone marrow mesenchymal stem cell
CCPP	copper-doped calcium polyphosphate
CPP	calcium polyphosphate
CPPC	calcium polyphosphate bone cement
CPP-MP	calcium polyphosphate microparticles
CPP-NP	calcium polyphosphate nanoparticles
COL-1	collagen type I
CS	chitosan
C_3_S	tricalcium silicate
CypB	cyclophilin B
Dex	dexamethasone
DOPA	dopamine
ERK	extracellular signal-regulated kinase
EuCPP	europium-doped calcium polyphosphate
FGF	fibroblast growth factor
HA	hydroxyapatite
HyA	hyaluronic acid
hBMSC	human bone marrow mesenchymal stem cell
HIF-1α	hypoxia-inducible factor 1 alpha
HUVEC	human umbilical vein endothelial cell
IκBα	inhibitor of nuclear factor kappa B alpha
iPSC	induced pluripotent stem cell
IP6K1/2	inositol hexakisphosphate kinase 1/2
LLPS	liquid–liquid phase separation
MFAT	microfragmented fat
MMP-1	matrix metalloproteinase 1
mPTP	mitochondrial permeability transition pore
NF-κB	nuclear factor kappa B
NPP-NP	sodium polyphosphate nanoparticles
OCN	osteocalcin
OPG	osteoprotegerin
OPN	osteopontin
PCL	polycaprolactone
Pi	inorganic phosphate
PLLA	poly(L-lactic acid)
PMMA	poly(methyl methacrylate)
PolyP	polyphosphate
PPIase	peptidyl-prolyl cis-trans isomerase
PPK	polyphosphate kinase
PVA	polyvinyl alcohol
RANKL	receptor activator of nuclear factor κB ligand
rGO	reduced graphene oxide
RSF	regenerated silk fibroin
Runx2	Runt-related transcription factor 2
SBF	simulated body fluid
SCPP	strontium-doped calcium polyphosphate
SDF-1	stromal cell-derived factor 1
SF	silk fibroin
SOST	sclerostin
Sox9	SRY-box transcription factor 9
3D	three-dimensional
TRAP	tartrate-resistant acid phosphatase
VEGF	vascular endothelial growth factor
XPR1	xenotropic and polytropic retrovirus receptor 1
